# Intolerance of uncertainty predicts fear of healthcare settings but not desire for testing during the novel coronavirus pandemic

**DOI:** 10.1007/s44202-021-00010-6

**Published:** 2021-12-20

**Authors:** Milen L. Radell, Brian M. McGuire

**Affiliations:** Department of Psychology, Niagara University, Lewiston, NY 14109 USA

**Keywords:** Anxiety, Avoidance, Coping, COVID-19, Fear, Individual differences, Personality

## Abstract

The novel coronavirus (COVID-19) pandemic has caused unprecedented uncertainty, and differences in how people cope with this uncertainty will influence the cost of viral pandemics to both individuals and society. The personality trait of intolerance of uncertainty (IU), defined as a dispositional fear of the unknown, has been linked to higher health anxiety and fear of the virus. Although IU may increase the desire for medical information and treatment, during pandemics, this might be weighed against the risk of becoming infected while in a healthcare setting. We examined whether people with higher IU report greater fear of healthcare settings, and show more desire to be tested for the virus. Residents of the United States (*n* = 149) were surveyed in early May 2020, while most states had active stay-at-home orders. Higher prospective but not inhibitory IU predicted more fear of healthcare settings. The largest effect size, however, was for fear of leaving the home, indicating a general tendency toward fear and avoidance. Fear of leaving the home, perceiving the virus as dangerous, access to testing, and having symptoms were significant predictors of desire for testing.

## Introduction

The novel coronavirus (COVID-19) pandemic has caused many around the world to face unprecedented uncertainty about the future. This includes uncertainty over whether we, or someone we know, has or will be infected by the virus, the course and severity of the illness, whether the vaccine and other prevention and treatment strategies will be effective, and the long-term impact on our health, income, and the country’s economy. Some people are better able to cope with this uncertainty than others. As such, there is a need to understand individual differences in coping, which will undoubtedly influence the cost of this, and other viral pandemics to both individuals and society. Of interest in our study is the personality trait of intolerance of uncertainty (IU), which can be defined as a dispositional fear of the unknown [1]. It is associated with aversion and avoidance of uncertain or ambiguous situations and has transdiagnostic relevance, having been linked to a number of psychological disorders, including anxiety and depression [2].

Even outside of any formal psychiatric diagnosis, IU may contribute to increased health anxiety [3], including during pandemics. For example, during the H1N1 pandemic in Canada, Taha et al. [4] found that higher IU predicted more anxiety over the virus, but this depended on the types of coping strategies people used. During the COVID-19 pandemic, Mertens et al. [5] found that IU was positively correlated with fear of the virus and health anxiety, and negatively correlated with overall health. This study was conducted in March 2020 on a sample from several different countries, predominantly from Europe but also North America [5]. Similarly, studies by Satici et al. [6] and Bakioğlu et al. [7] found a positive correlation between IU and fear of COVID-19 in samples from Turkey. Higher IU also predicted more depression, anxiety and stress, and lower positivity, defined as an optimistic emotional state or attitude [7]. IU was also negatively correlated with mental wellbeing. This relationship, however, depended on the type of coping strategy used and fear of the virus [6].

In a Greek sample, surveyed in April 2020, higher IU also predicted more depression symptoms, a relationship that also depended, in part, on fear of COVID-19 [8]. In a sample from Argentina, IU was also associated with more depression and anxiety symptoms, especially in women, though this gender difference was small [9]. In a Romanian sample, IU was a significant predictor of perceived risk of COVID-19, but was not related to compliance with preventive measures [10]. IU was also positively correlated with concern and fear of the virus in a sample from the United States, surveyed in March 2020 [11]. In a sample from the United Kingdom, IU was related to psychological distress, health anxiety, and depression during the COVID-19 pandemic [12]. In United States residents surveyed mid-April to early May 2020, there was a positive correlation between social isolation and anxiety, but only in those with higher IU [13]. In other samples from the United States, higher IU was related to more fear of the virus, independent of general anxiety and depression levels [14], as well as with health anxiety and greater perceived likelihood and negative consequences of the virus [15]. However, a study by Sauer et al. [16] conducted in Germany found no evidence that IU moderates the relationship between pre-pandemic health anxiety, measured retrospectively, and health anxiety during the pandemic. In summary, most studies, across multiple geographic regions, have found that IU is related to several mental health outcomes during the COVID-19 pandemic.

One way to cope with the uncertainty associated with a potential threat, such as that of a virus, is to seek more information. Thus, it is reasonable to expect that IU is related to increased demand for medical information, testing and treatment as a way to reduce uncertainty. Some of the past research in this area has examined whether IU is related to hypochondriasis [17]. This disorder involves inaccurate beliefs about health status, and can be seen as an extreme form of health anxiety [18]. It features the tendency to misinterpret various body sensations as symptoms of a serious medical illness. For example, a headache might be seen as indicative of a brain tumor [18]. Interestingly, while hypochondriasis is associated with a desire for frequent medical testing, inaccurate beliefs persist even after the tests come back negative [18]. It is common for people with medical concerns, including those with hypochondriasis, to look up and collect medical information on the internet. For some, this can backfire and increase health anxiety, possibly because it is easy to misinterpret harmless body sensations as the symptoms of serious, but relatively rare, medical conditions listed online [3]. Consistent with this, Fergus and Bardeen [3] found that people with higher IU search for more medical information, likely as a way to find reassurance that nothing is wrong and reduce anxiety.

A clear prediction from this type of research is that, during a pandemic, higher IU should be associated with increased motivation for medical testing, in particular to discover if one has been infected by the virus. From a public health standpoint, testing is critical for identifying locations where a virus is more widespread, and for contact tracing, to slow transmission. It has also been proposed as a way to balance public health and economic concerns (e.g., as a way to participate in large social gatherings or other activities) [19]. However, relatively few studies have examined what factors influence people’s willingness to be tested for COVID-19 [19–21]. They include demographic factors, such as race and ethnicity, socioeconomic status, and education, as well as concern about the virus, the desire to avoid infecting others, and the perceived costs of getting tested, such as having to quarantine and miss work if the test is positive [19]. A main goal of our study was to extend this research by examining whether IU is also related to the desire or willingness to get tested.

In addition to getting tested, people can cope with the health anxiety brought about by pandemics through a variety of other behaviors, including hygiene-related behaviors (e.g., washing hands, disinfecting surfaces) and avoidance-related behaviors (e.g., cancelling social events or travel plans) [22]. During the COVID-19 pandemic, Seale et al. [22] found that people tended to engage in more avoidance-related behaviors if they had more trust in the government or authorities, and perceived these behaviors as effective and practically feasible. Of interest in our study is fear and avoidance of healthcare settings. This can be expressed in a number of ways, including cancelling medical appointments and avoiding doctor’s offices.

Based on data from the Centers for Disease Control, the number of emergency department visits in the early period of the pandemic (i.e., end of March to late April 2020) was 42% lower compared to the same time the previous year. The largest declines were for children under the age of 15, women, and those in the northeast United States, including the region with New York and New Jersey [23]. Similar trends were observed elsewhere. For example, in a survey conducted in the United Kingdom and Ireland, physicians reported a decrease in children visiting emergency departments during the pandemic, with at least some patients coming later than they should have [24]. Lazzerini et al. [25] reported a major decrease in pediatric emergency department visits during Italy’s national lockdown in March 2020, compared to the same time the previous two years. Although some of this could be due to a decrease in infections or injuries related to the closure of schools, and the cancellation of sporting events, at least some of this decline was due to parents avoiding taking their children to the hospital out of fear of COVID-19 infection [25]. Mantica et al. [26] compared emergency department visits during the pandemic for two hospitals in Italy, focusing on late February to mid-April. The lowest point of visits coincided with the highest point in COVID-19 mortality [26]. In a study of a hospital in California, Wong et al. [27] reported a decrease in visits (reaching about 50%) from March 20 when a shelter-in-place order went into effect in the state, until late April, compared to the same time period the previous year. At the same time, the highest ever number of cardiac arrests were reported, suggesting that at least some waited too long to seek medical attention [27]. Based on semi-structured interviews to understand the reasons for the decline in visits, patients tended to report being afraid of going to the hospital, which was seen as a risky location, and uncertainty about the precautions taken to mitigate the risk of COVID-19 infection, including what to expect once inside [27].

Thus, while higher IU may increase the desire for medical testing and treatment, during pandemics, this desire might be weighed against the risk of becoming exposed and infected while in healthcare settings. Although a number of studies cited above have related IU to fear of the virus itself, they did not specifically address fear of healthcare settings and the desire to be tested for the virus. We sought to extend this research by examining two hypotheses:

1. People with higher IU would be more afraid of going to the doctor, or healthcare settings in general.
2. Those with higher IU would show a greater desire to be tested for the virus.

This study adds to the knowledge of how IU affects people’s responses to pandemics. It is also relatively unique in that data were collected from residents of the United States near the end of April of 2020, while the pandemic was still relatively new, and while most states had active stay-at-home orders.

## Methods

We report all measures and data exclusions, and provide the study materials and data on the Open Science Framework website (https://osf.io/f2g3k/).

### Participants

To determine sample size, we conducted an a priori power analysis in GPower (version 3.1) based on one of the few studies on fear of COVID-19 available at the time. Specifically, Mertens et al. [5] found a correlation between fear of COVID-19 and IU of *r* = 0.27, which represents a small to medium effect size. We assumed that we would also find a relatively small to medium (Cohen’s f^2^ = 0.10) effect size for the relationship between IU and fear of healthcare settings. With an alpha level of 0.05, a total sample size of 134 is sufficient to achieve 80% power in a multiple regression with five predictors. We tested an additional 10–15% to account for the possibility of excluding participants due to incomplete survey responses, and for including more predictors in the regression.

Thus, a total of 150 participants were recruited from the online research platform Prolific (https://www.prolific.co/) who were compensated $1.75 for completing the study. To participate, individuals had to be at least 18 years old and residing in the United States. At the time, Prolific found a total of 30,089 people who matched these criteria. On Prolific, participants saw the following study description: “The general purpose of the study is to examine people’s experiences during the novel coronavirus (COVID-19) health crisis and whether they are related to personality. You will be asked to provide demographic information and fill out some questionnaires”. Informed consent was provided before any study procedures began. The procedures were approved by the Niagara University Institutional Review Board and conform to the U.S. Federal Policy for the Protection of Human Subjects.

### Procedure

Participants completed a survey that included items to assess fear of healthcare settings during the COVID-19 pandemic, the desire to get tested for the virus, and IU, as detailed below. They were also asked to provide demographic information. The survey was administered online via SurveyMonkey (https://www.surveymonkey.com/) and could be completed through a personal computer, smart phone or tablet. Data collection started on Saturday, May 5, 2020 at 7 PM Eastern Standard Time, and was complete by the following morning.

#### Fear of healthcare settings

To assess fear and avoidance of healthcare settings, participants responded to a custom-built questionnaire, consisting of the following items:

1. I would be afraid of going to a doctor’s office because I might get infected by the coronavirus.
2. I would choose to cancel or reschedule an important medical appointment because of the coronavirus.
3. If I have medical concerns I would be reluctant to go to the doctor because it’s dangerous there due to the coronavirus.
4. I would feel safe going to a hospital or other healthcare setting during the coronavirus health crisis.

The response options were on a 6-point Likert scale (from 1 = “Strongly disagree” to 6 = “Strongly agree”). The questions were chosen to focus on avoidance of healthcare settings as a behavioral expression of fear. The internal consistency of the items, based on Cronbach’s alpha, was 0.90 in the sample, with an average inter-item correlation of 0.68. For analysis, we computed the sum of the individual items, with item 4 reverse scored, so that a higher total score indicated more fear of healthcare settings.

#### Fear of COVID-19

Since fear of healthcare settings should be related to fear of the virus itself, we were also interested in measuring it. To do so, we modified a single item from the fear of COVID-19 questionnaire developed by Mertens et al. [5], namely, “For my personal health I find the coronavirus to be dangerous”. The original question from Mertens et al. [5] asked participants whether they thought the coronavirus was much more dangerous than the seasonal flu for their personal health. In addition, we asked the following: “I’m afraid of leaving my home because I might get infected by the coronavirus”. This question was included to establish whether any fear of healthcare settings is accompanied by a more general fear of leaving the home.

#### Desire for testing

We also assessed the desire to get tested for the coronavirus because testing provides a way to reduce uncertainty. Therefore, those with higher IU might express a stronger desire for testing, which was measured through another custom-built questionnaire, consisting of the following items:

1. Even if I have no symptoms, I would still like to be tested for the coronavirus.
2. It’s important for me to know if I’ve had the coronavirus.
3. I would like to be tested regularly to check if I have the coronavirus.
4. Testing to see if someone has the coronavirus is important.

The response options were on a 6-point Likert scale (from 1 = “Strongly disagree” to 6 = “Strongly agree”). The internal consistency of the items, based on Cronbach’s alpha, was 0.77, with an average inter-item correlation of 0.46. For analysis, the sum of the individual items was computed so that a higher total score indicated more desire for testing.

#### Intolerance of uncertainty

We used the brief 12-item version of the Intolerance of Uncertainty Scale (IUS-12) [28] to measure this construct. This measure has two subscales, one related to anxiety due to uncertainty about the future, which we will refer to as prospective IU, and one related to the ability to act in the face of uncertainty—inhibitory IU [28]. Sample items include “Unforeseen events upset me greatly” for prospective IU, and “The smallest doubt can stop me from acting” for inhibitory IU. The response options range from 1 = “Not at all characteristic of me” to 5 = “Entirely characteristic of me”. As reviewed earlier, most prior studies of IU during the COVID-19 pandemic only considered total IU. Instead, we opted to examine prospective and inhibitory IU separately. In our sample, the internal consistency of the entire scale based on Cronbach’s alpha was 0.87, while that for the prospective and inhibitory IU subscales was 0.72 and 0.86, respectively. The average inter-item correlations were 0.34 for the entire scale, 0.27 for prospective IU, and 0.54 for inhibitory IU.

#### Demographics

Since it was already well-known that age is a risk factor for virus-related complications, participants were asked to provide their age. They were also asked about their gender, race, whether they identified as Hispanic or non-Hispanic, highest level of education obtained, whether they are an essential worker, a first responder (e.g., healthcare professional, police officer, firefighter), whether they work in a healthcare setting (e.g., doctor’s office, hospital, nursing home) and if they have been able to work from home during the coronavirus health crisis (ranging from “Never” to “Always”). We also asked what state they live in because different states had different infection rates, as well as different adherence to mitigation strategies or guidelines for dealing with the virus, at the time of data collection (i.e., April 30, 2020). Additionally, we asked if participants were experiencing symptoms that they think might be due to the coronavirus, whether they had been tested for the virus, and if so, whether they had ever tested positive. Two questions that dealt with perceived access to healthcare and to COVID-19 testing, respectively, were also included.

### Data analysis

The statistical analyses were done in R (version 4.1.0) and a *p* < 0.05 was considered statistically significant. All tests were two-tailed.

To test our first hypothesis, we conducted a multiple regression with a forced entry method on fear of healthcare settings, with total score on the prospective and inhibitory subscales of the IUS-12. The models also included age, gender, perceived danger of the virus, and fear of leaving the home. We considered age and gender because they were already known to predict fear of the virus [5, 6]. Those who perceive the virus as more dangerous to their personal health can also be expected to avoid healthcare settings, due to fear of infection. We also included fear of leaving the home as a measure of more general fear and avoidance.

A similar analysis was conducted to test the second hypothesis about desire for testing. The model included the same predictors as above, in addition to perceived access to testing and whether participants reported experiencing symptoms that might be due to COVID-19, which could both contribute to the motivation to get tested.

As a measure of effect size, we computed standardized beta values (β) by transforming the outcome variable, and all continuous predictors, into z-scores using the *dplyr* package in R. The regression was then rerun. These values represent the number of standard deviations by which the outcome variable is estimated to change due to one standard deviation change in the predictor, while holding the effects of all other variables constant.

The assumptions of multiple regression were assessed as described in Field et al. [29]. Briefly, to test for multicollinearity, variance inflation factor (VIF) and tolerance statistics were computed. The VIF for each predictor is given by the *vif* function from the *car* package, while tolerance is equal to 1/VIF. We also computed the mean VIF for the model. The assumption of independent errors was assessed via the Durbin-Watson test, using the *dwt* function from the *car* package in R. To check other assumptions about the residuals, we examined Q-Q plots, and plots of the residuals against fitted values for each model.

## Results

### Demographics

One participant was excluded from analyses due to incomplete survey responses. The final sample included 149 people (79 females, 53.02%), with a mean age of 30.58 years (ranging 18–71, *SD* = 10.87). The sample was primarily white (101 people, 67.79%), with 20 identifying as Asian, 13 as African American, 10 were classified as having Mixed race due to selecting more than one racial category, 3 chose Other and 2 selected Native Hawaiian or Other Pacific Islander. When asked about their ethnicity, only 13 (8.73%) identified as Hispanic, while 2 (1.34%) preferred not to answer. Most of the sample (129 people, 86.58%) had completed at least some college, while the rest reported completing high school or an equivalent. Specifically, with respect to the highest level of education achieved, 20 reported graduating from high school or equivalent, 14 completed 1 year of college, 17 completed 2 years of college, another 17 reported 3 years of college, 60 obtained a Bachelor’s degree, 5 some graduate school but no degree, 12 a Master’s degree, and 4 a doctoral or professional degree. A total of 26 people (17.45%) identified as essential workers, but only 8 (5.37%) stated that they work in a healthcare setting, and 2 (1.34%) reported being first responders. With respect to working from home during the pandemic, the most common responses were at the extremes—49 (32.89%) reported never being able to work from home while 51 (34.23%) could always work from home. A majority (67.79%) tended to at least slightly agree with having access to healthcare. Only 7 (4.70%) had been tested for COVID-19, and none reported testing positive. Participants resided in a variety of states, but the highest number (25 people, 16.78%) were from California. Descriptive statistics for continuous variables are shown in Table 1.

**Table 1:** Descriptive statistics for continuous variables (n = 149)

	M	SD	Min.	Max.
Fear healthcare	15.09	5.17	4	24
Desire testing	16.63	4.10	5	24
Age (in years)	30.58	10.87	18	71
Total IU	35.19	8.03	14	51
Prospective IU	21.97	4.38	9	32
Inhibitory IU	13.22	4.38	5	24
Fear leaving home^a^	3.49	1.39	1	6
Virus dangerous^b^	4.01	1.46	1	6
Testing access^c^	2.73	1.57	1	6
Have symptoms^d^	1.56	0.81	1	4

IU, intolerance of uncertainty

^a^I’m afraid of leaving my home because I might get infected by the coronavirus

^b^For my personal health I find the coronavirus to be dangerous

^c^I think healthcare authorities have provided adequate access to testing for the coronavirus

^d^I’m experiencing symptoms that I think might be the coronavirus

### Fear of healthcare settings

The results of the multiple regression for fear of healthcare settings are shown in Table 2. The model was significant, *F*(6, 142) = 12.85, *p* < 0.001, multiple *R*^*2*^ = 0.35, *R*^*2*^_*adj*_ = 0.32. The correlation between prospective and inhibitory IU was high (Pearson’s *r* = 0.92), raising possible concerns about multicollinearity in the model. As described in the Data Analysis section, we computed VIF and tolerance statistics to assess the degree of multicollinearity. The highest VIF was 1.91 and the lowest tolerance was 0.52, both for prospective IU, while the mean VIF for the model was 1.48. As a general rule, a VIF much greater than 1, and a tolerance less than 0.2 are cause for concern [29]. Thus, the degree of multicollinearity in this model was still relatively low. The assumption of independent errors (Durbin-Watson value = 1.77, *p* = 0.122), and the remainder of the assumptions about the residuals were also satisfied.

**Table 2:** Summary of multiple regression for variables predicting fear of healthcare settings (n = 149)

Included	*B* (SE)	95% CI for *B*		β	*T*	*P*
		Lower	Upper			
Constant	0.63 (2.25)	− 3.82	5.09		0.28	0.780
Age (in years)	0.09 (0.03)	0.02	0.16	0.19	2.72	0.007
Gender (female)	0.72 (0.70)	− 0.67	2.11		1.02	0.308
Prospective IU	0.32 (0.08)	0.10	0.53	0.27	2.87	0.004
Inhibitory IU	− 0.21 (0.11)	− 0.42	0.01	− 0.17	− 1.88	0.062
Virus dangerous^a^	0.35 (0.29)	− 0.23	0.93	0.10	1.19	0.237
Fear leaving home^b^	1.62 (0.30)	1.02	2.22	0.44	5.36	< 0.001

Model *F*(6, 142) = 12.85, *p* < 0.001, multiple *R*^*2*^ = 0.35, *R*^*2*^_*adj*_ = 0.32

IU, intolerance of uncertainty

^a^For my personal health I find the coronavirus to be dangerous

^b^I’m afraid of leaving my home because I might get infected by the coronavirus

**Fig. 1:**
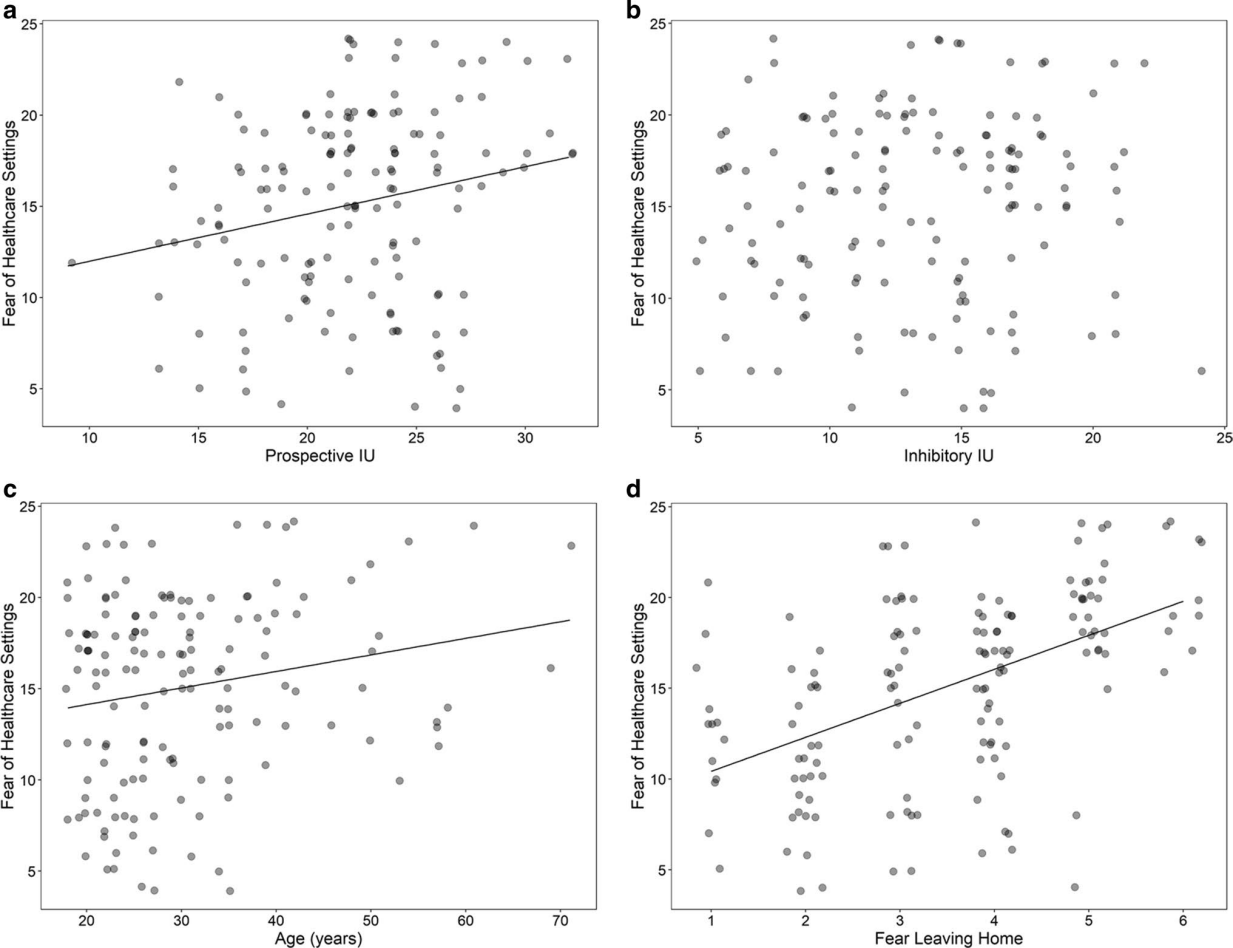
Predictors for fear of healthcare settings. **a** Prospective IU was significantly related to fear of healthcare settings. **b** No relationship was found with inhibitory IU. **c** Older participants reported more fear of healthcare settings. **d** Fear of leaving the home was also significant, suggesting a more general tendency toward fear and avoidance, that is not limited to healthcare settings

In partial support of our first hypothesis, higher prospective, but not inhibitory IU, predicted more fear of healthcare settings (Fig. 1A and B, respectively). As expected, age was also significant, with older participants reporting more fear of healthcare settings (Fig. 1C). The strongest predictor, however, was fear of leaving the home (Fig. 1D). Surprisingly, perceiving the virus as dangerous was not significant. There was also no evidence for a gender difference in fear of healthcare settings, which had a mean of 14.43 (*SD* = 4.57) for males and 15.68 (*SD* = 5.60) for females.

### Desire for testing

The results of the multiple regression for desire for testing are shown in Table 3. The model was significant, *F*(8, 140) = 8.497, *p* < 0.001, multiple *R*^*2*^ = 0.33, *R*^*2*^_*adj*_ = 0.29. The highest VIF was 1.96, and the lowest tolerance was 0.51, both for prospective IU. The mean VIF for the model was 1.39. Thus, the degree of multicollinearity in this model was relatively low. The assumption of independent errors (Durbin-Watson value = 1.96, *p* = 0.784), and the remainder of the assumptions about the residuals were also satisfied.

**Table 3:** Summary of multiple regression for variables predicting desire for testing (n = 149)

Included	*B* (SE)	95% CI for *B*		β	*T*	*P*
		Lower	Upper			
Constant	11.54 (1.93)	7.72	15.37		5.97	< 0.001
Age (in years)	− 0.02 (0.03)	− 0.08	0.03	− 0.06	− 0.87	0.385
Gender (female)	− 0.19 (0.59)	− 1.35	0.96		− 0.33	0.741
Prospective IU	0.00 (0.09)	− 0.18	0.18	0.00	0.02	0.982
Inhibitory IU	− 0.00 (0.09)	− 0.18	0.18	− 0.00	− 0.02	0.986
Virus dangerous^a^	1.05 (0.24)	0.58	1.52	0.37	4.39	< 0.001
Fear leave home^b^	0.51 (0.25)	0.02	1.00	0.17	2.06	0.041
Testing access^c^	− 0.50 (0.19)	− 0.87	− 0.12	− 0.19	− 2.64	0.009
Have symptoms^d^	0.81 (0.36)	0.11	1.52	0.16	2.29	0.023

*Note.* Model *F*(8, 140) = 8.497, *p* < 0.001, multiple *R*^*2*^ = 0.33, *R*^*2*^_*adj*_ = 0.29

IU, intolerance of uncertainty

^a^For my personal health I find the coronavirus to be dangerous

^b^I’m afraid of leaving my home because I might get infected by the coronavirus

^c^I think healthcare authorities have provided adequate access to testing for the coronavirus

^d^I’m experiencing symptoms that I think might be the coronavirus

**Fig. 2:**
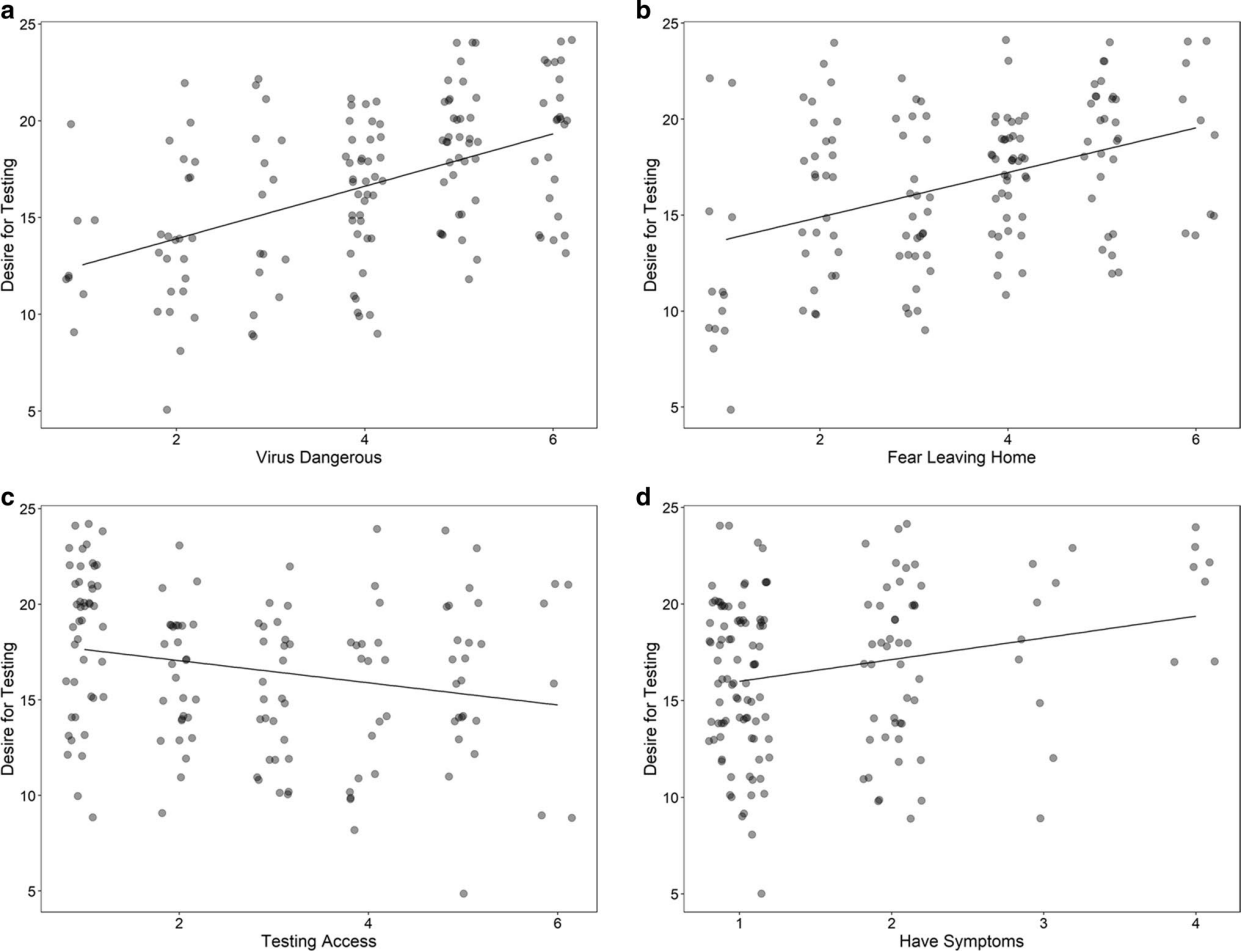
Predictors for desire for testing. **a** Perceiving the virus as dangerous to one’s own personal health, as well as **b** fear of leaving the home were associated with greater desire for testing. **c** Participants who reported more desire for testing were also more likely to disagree that healthcare authorities had provided adequate access to testing. **d** As expected, agreeing with having symptoms that might be due to COVID-19 was associated with more desire for testing. However, most participants in our sample tended to disagree with this statement

Contrary to our second hypothesis, prospective and inhibitory IU were not related to desire for testing. Similarly, there was no relationship with age or gender—mean desire for testing was 16.51 (*SD* = 3.77) for males, and 16.73 (*SD* = 4.38) for females. However, perceiving the virus as dangerous (Fig. 2A), being afraid of leaving the home (Fig. 2B), and reporting having symptoms that might be due to COVID-19 (Fig. 2D), all significantly predicted greater desire for testing. In addition, those who had a greater desire for testing were less satisfied with the availability of testing (Fig. 2C).

## Discussion

In partial support of our first hypothesis, higher prospective IU predicted more fear of healthcare settings (Fig. 1A). This relationship, however, was relatively weak. Surprisingly, no relationship was found between fear of healthcare settings and inhibitory IU (Fig. 1B). Why might this be? Since prospective IU deals with anxiety due to uncertainty about the future, it might be more important when thinking about whether to visit a healthcare setting, and whether doing so would be safe during a pandemic. Freeston et al. [2] define IU as a tendency to be bothered or upset by an uncertain situation, regardless of what the possible outcome of the situation is (i.e., it does not matter if it is a positive or negative outcome). They propose IU leads people to experience uncertain situations as aversive, perform behaviors that help relieve the distress associated with uncertainty, and can also influence their perception of how uncertain and threatening the situation is [2]. In a pandemic, going to a healthcare setting might help reduce uncertainty due to medical concerns (e.g., personal, those of a child or other loved one), but this would also have to be weighed against the uncertainty associated with the possibility of becoming infected.

Mertens et al. [5] and Satici et al. [6] found that higher total IU was associated with more fear of COVID-19, but did not consider the two dimensions of IU separately. They both measured IU via the IUS-12, as we did. In addition, in Mertens et al.’s study [5], IU was no longer significant when included in a regression with other predictors of fear of the virus. Instead, perceived risk for loved ones, exposure to the media, and health anxiety were related to fear of COVID-19 [5]. Although fear of the virus could contribute to fear of healthcare settings, it is nonetheless a separate construct, and in our sample, perceiving the virus as dangerous was not related to fear of healthcare settings when accounting for other variables (Table 2). It is possible that other concerns, not measured in our study, also contribute. For example, Mertens et al. [5] found that the health of others (e.g., friends or relatives) was reported as a major concern by 46% their sample. In comparison, personal health was mentioned by only 11% of participants. Therefore, fear of healthcare settings could also be related to a concern for others.

Fear of leaving the home was also related to fear of healthcare settings (Fig. 1D), and had the largest effect size (Table 2). This suggests that there was a more general tendency toward fear and avoidance, not limited to healthcare settings. Age was also a significant predictor (Fig. 1C), which was not surprising, given that both healthcare authorities and media coverage, starting from the beginning of the pandemic, consistently focused on the threat the virus poses to the elderly. It is also consistent with Mertens et al. [5] who found that older individuals tended to perceive COVID-19 as more dangerous than the flu and were more afraid of it. That study also found that media exposure predicted fear of COVID-19, an issue that has been more extensively discussed by others, both in the context of the current as well as previous pandemics [30]. Although media exposure can increase fear, as Garfin et al. [30] point out, it can also promote protective measures (e.g., wearing a mask, washing hands, social distancing, etc.).

Our second hypothesis that people with higher IU would also show a greater desire for testing was not supported. Testing is one way to reduce uncertainty about whether or not one is infected, therefore, this was surprising. Instead, the significant predictors of desire for testing included perceiving the virus as dangerous to one’s own personal health (Fig. 2A), which had the largest effect size (Table 3), and is consistent with other research [19]. Fear of leaving the home, perceived access to testing, and reporting having symptoms that could be due to COVID-19, were also significant predictors (Fig. 2B, C, D). The relationship between desire for testing and perceived access to testing was negative, indicating that those who had a stronger desire for testing also tended to be less satisfied with the availability of testing (Fig. 2C).

Why did we fail to find a relationship between IU and desire for testing? This is unclear. It has been proposed that prospective and inhibitory IU may contribute to the IU construct in opposite directions. For example, prospective IU should be associated with a desire for a predictable world and motivate collecting information to reduce uncertainty [31]. This could lead to over-engagement in the form of over-preparation, repeated questioning and more internet searching, which can all be thought of as approach behaviors [2]. From this perspective, higher prospective IU should have been associated with more desire for testing. In contrast, inhibitory IU should be associated with avoidance, involving disengaging or distancing from an uncertain situation, which can help reduce the distress caused by that situation. This could involve procrastination, distraction and avoidance of information [2] and predicts a negative relationship with desire for testing. However, people with higher IU can use both of these strategies, switch between them, or pursue neither due to indecisiveness, resulting in inaction [2]. In a pandemic, there could also be risk associated with going to a testing site, which could also be a healthcare setting, or perceived to be just as dangerous. This could help explain why even people with higher IU might not want to get tested. More research is needed to understand how IU interacts with perceived and actual threat to influence behavior.

In the Freeston et al. [2] model, distress associated with uncertainty can result from perceived and actual threat, perceived and actual uncertainty, as well as dispositional and situational IU, where dispositional IU (in our study, measured by the IUS-12) is related to situational IU. In addition, the levels of perceived uncertainty and threat can both depend on dispositional IU. In a pandemic, there is actual threat, while in the context of anxiety disorders, where IU has been most well-studied, there tends to be high perceived threat and low actual threat [2]. For this reason, rather than labeling the distress experienced during a pandemic as a disorder, it should be understood as an appropriate reaction to the situation. Freeston et al. [2] argue for trying to partition how threat and uncertainty contribute to distress. It can help clients or patients understand what is real and what is perceived, and identify contributors that can be targeted by specific treatment strategies to reduce distress.

A limitation of our study was reliance on self-report. However, a strength was that the data were collected relatively early in the pandemic, while most states were under stay-at-home orders. Thus, participants provided current, rather than retrospective reports, which should increase the validity of the results. Nonetheless, the participants were still asked about what they would do (e.g., would you cancel a medical appointment?), not about what they have already done (e.g., have you cancelled a medical appointment?). Thus, it is unclear how many would have actually avoided healthcare settings, especially when faced with a serious medical problem. At this point, the fear of what might happen if they do not seek medical attention might overwhelm any concerns about contracting the virus. Thus, future studies of behavior during pandemics might consider more specific questions (e.g., number of appointments people have cancelled, number of visits to healthcare settings). The same applies to desire for testing—reporting more desire to be tested may not correspond to actual behavior, especially with a general fear of leaving the home due to possible risk of infection.

Furthermore, the results we report are correlational, and do not establish cause and effect relationships between any of the variables considered. The study was also cross-sectional, although this was at a time, relatively early in the pandemic, when situational uncertainty should have been high. It is possible that IU may have contributed to behavior during the pandemic in different ways at different times. One longitudinal study in college students suggests that dispositional IU remained relatively stable, with moderate correlations (close to 0.50) for prospective and inhibitory IU measured at three different time points, including just before and during the COVID-19 pandemic [32]. Nonetheless, as the model of Freeston et al. [2] would predict, the effect of dispositional IU may have changed as the uncertainty associated with the situation changed (i.e., as more became known about COVID-19). Lastly, our sample was not representative of the general population in the United States, given that recruitment was through an online research platform. This is also reflected in the age and other demographic characteristics of our sample, which was biased in favor of younger college-educated adults.

In summary, we found that fear of leaving the home was a relatively strong predictor of both fear of healthcare settings and desire for testing. In addition, prospective but not inhibitory IU was related to fear of healthcare settings. Neither were related to desire for testing. With hospitals perceived as high-risk locations [27], information about the measures that have been taken to mitigate risk could help reassure people that it is relatively safe to seek medical care, particularly when there is an issue that may require emergency treatment. This information can be made public through various channels (e.g., provider website, social media, pre-recorded messages to patients, etc.), which could help reduce the uncertainty and fear associated with visiting a healthcare setting. Wong et al. [27] provide examples of simple messaging, focused on measures such as temperature and symptom screening, separating patients with symptoms that could indicate infection (e.g., respiratory symptoms) from other visitors, masks worn by all staff and provided to all patients, enhanced cleaning procedures, and inpatient visitor limits. Patients could also wait outside or in their car to be called in, instead of in a common waiting room. Importantly, regardless of what measures are taken, they should be clearly communicated to patients ahead of time.

## Data Availability

The data are available on the Open Science Framework (https://osf.io/f2g3k/).
